# Histone Deacetylase Inhibitor Upregulates Peroxisomal Fatty Acid Oxidation and Inhibits Apoptotic Cell Death in Abcd1-Deficient Glial Cells

**DOI:** 10.1371/journal.pone.0070712

**Published:** 2013-07-26

**Authors:** Jaspreet Singh, Mushfiquddin Khan, Aurora Pujol, Mauhamad Baarine, Inderjit Singh

**Affiliations:** 1 Department of Pediatrics, Darby Children Research Institute, Medical University of South Carolina, Charleston, South Carolina, United States of America; 2 Neurometabolic Diseases Laboratory, Institute of Neuropathology, Bellvitge Institute for Biomedical Research (IDIBELL), Hospitalet de Llobregat, Barcelona, Spain; 3 Center for Biomedical Research on Rare Diseases (CIBERER), Barcelona, Spain; 4 Catalan Institution for Research and Advanced Studies (ICREA), Barcelona, Spain; Friedrich-Alexander University Erlangen, Germany

## Abstract

In X-ALD, mutation/deletion of ALD gene (ABCD1) and the resultant very long chain fatty acid (VLCFA) derangement has dramatically opposing effects in astrocytes and oligodendrocytes. While loss of Abcd1 in astrocytes produces a robust inflammatory response, the oligodendrocytes undergo cell death leading to demyelination in X-linked adrenoleukodystrophy (X-ALD). The mechanisms of these distinct pathways in the two cell types are not well understood. Here, we investigated the effects of Abcd1-knockdown and the subsequent alteration in VLCFA metabolism in human U87 astrocytes and rat B12 oligodendrocytes. Loss of Abcd1 inhibited peroxisomal β-oxidation activity and increased expression of VLCFA synthesizing enzymes, elongase of very long chain fatty acids (ELOVLs) (1 and 3) in both cell types. However, higher induction of ELOVL's in Abcd1-deficient B12 oligodendrocytes than astrocytes suggests that ELOVL pathway may play a prominent role in oligodendrocytes in X-ALD. While astrocytes are able to maintain the cellular homeostasis of anti-apoptotic proteins, Abcd1-deletion in B12 oligodendrocytes downregulated the anti-apototic (Bcl-2 and Bcl-xL) and cell survival (phospho-Erk1/2) proteins, and upregulated the pro-apoptotic proteins (Bad, Bim, Bax and Bid) leading to cell loss. These observations provide insights into different cellular signaling mechanisms in response to Abcd1-deletion in two different cell types of CNS. The apoptotic responses were accompanied by activation of caspase-3 and caspase-9 suggesting the involvement of mitochondrial-caspase-9-dependent mechanism in Abcd1-deficient oligodendrocytes. Treatment with histone deacetylase (HDAC) inhibitor suberoylanilide hydroxamic acid (SAHA) corrected the VLCFA derangement both *in vitro* and *in vivo*, and inhibited the oligodendrocytes loss. These observations provide a proof-of principle that HDAC inhibitor SAHA may have a therapeutic potential for X-ALD.

## Introduction

The ALD gene (ABCD1), identified by positional cloning [1], encodes a protein ALDP that is related to the peroxisomal ATP-binding cassette (ABCD) transmembrane transporter proteins [2], [3]. Loss of ABCD1 function results in defective β-oxidation of very long chain fatty acids (VLCFA) [4] resulting in accumulation of VLCFA, the biochemical “hallmark” of X-ALD, in plasma and tissues, most notably in brain and adrenal cortex [5]. Frequently clinically distinct phenotypes ranging from a fatal childhood cerebral ALD (cALD) to relatively benign adult disease of adrenomyeloneuropathy (AMN) occur within the same family with no phenotype-genotype correlation having been established so far. The molecular events that trigger the transition from the metabolic derangement, common to all forms of X-ALD, to neuroinflammation and demyelination in cALD or to axonal degeneration in spinal cords in AMN are largely unknown. Recent studies from our laboratory [6], [7] and others [8] show a correlation between VLCFA accumulation caused by silencing of peroxisomal transporters in neural tissue in X-ALD and glial cells to redox imbalance, and changes in membrane lipid composition [6], [7], [9], [10], [11] leading to astrocytic inflammatory response and loss of oligodendrocytes and myelin [10], [12].

Abcd1 knockout mouse does not develop demyelination characteristic of cALD, although myelin disturbances are evident starting at 15-month in sciatic nerve and spinal cord tissue [13], although it does show nuclear factor-κB (NFkB) pro-inflammatory cytokine induction [14]. In active lesions of X-ALD brain, astrocytes expressed large amounts of tumor necrosis factor-α (TNF-α) [12] and inducible nitric oxide synthase (iNOS) [15]. Since acute glial death is reported to promote neuronal death [16], the glial loss in X-ALD probably plays a role in the progression of neurodegeneration in X-ALD. The recently reported differential accumulation of VLCFA in induced pluripotent stem cell (iPSC)-derived oligodendrocytes from X-ALD and AMN fibroblasts [17] suggests that Abcd1 loss may induce different cellular signaling or metabolic derangements in these cell types.

In addition to β-oxidation defect, increased expression of elongases (ELOVLs) also contributes to higher VLCFA levels [18]. However, the effect of Abcd1-deletion on ELOVLs in astrocytes and oligodendrocytes has not been explored. Moreover, inflammatory mediators (TNF-α and IL-1β) downregulate peroxisomal β-oxidation function [19]. Accordingly, different degrees of VLCFA accumulation were observed in different areas (inflammatory, plaque and non-inflammatory) of X-ALD brain. In X-ALD CNS, therefore, altered activities of ELOVLs and peroxisomal β-oxidation as well as the secondary effects of inflammatory mediators may contribute towards the observed pathognomic levels of VLCFA. Hence, an effective therapy should be able to correct the metabolic derangements as well as attenuate the inflammatory responses.

Current treatment options for X-ALD are limited [5]. The concept of therapeutic induction of functionally redundant Abcd2/Abcd3 has initiated intense investigations aiming to modulate the ABCD gene expression as a novel therapeutic strategy for X-ALD. Previous studies from our laboratory and others have demonstrated that Abcd2 expression could be upregulated in rodents by various therapeutic compounds [17], [20], [21], [22], but no induction was found in brain for various reasons [23]. Long-term treatment with 4-phenylbutyrate in Abcd1-KO mouse model lead to a reduced drug response and VLCFA levels returned to pretreatment levels [24]. Valproic acid (VPA) induced the Abcd2 expression but was unable to lower the levels of VLCFA [20]. We have formerly shown that lovastatin and sodium phenylacetate, can enhance VLCFA β-oxidation and reduce VLCFA levels in human skin fibroblasts [25], lymphoblasts [26] and plasma of X-ALD patients [27].

Using U87 astrocytes and B12 oligodendrocytes stably silenced for Abcd1 using lentiviral vectors, this study describes the astrocyte vs. oligodendrocyte-specific VLCFA-mediated derangements and activation of mitochondrial cell death pathways. We also evaluated the effect of SAHA treatment in these cells. Treatment with SAHA corrected the metabolic derangements as well as inhibited the Abcd1-deficiency-induced apoptotic response. Additionally, the effect of SAHA was investigated in hippocampal slice cultures from patients suffering from drug-resistant epilepsy that were scheduled for hippocampal resection, where it upregulated the ABCD2 expression. Remarkably, Abcd1-KO mice treated with SAHA in diet had significantly lower VLCFA levels in the brain providing the first pre-clinical proof-of-principal for testing SAHA as therapy for X-ALD.

## Experimental Procedures

### Ethics Statement

Hippocampal specimens were derived from three patients submitted to epilepsy surgery at the Erlangen Epilepsy Center. The patients suffered from drug-resistant epilepsy and were scheduled for hippocampal resection. For scientific use of tissue specimens, written informed consent was obtained from each patient with the approval of the local ethics committee of the University Hospital of Erlangen.

### Reagents

Dulbecco's modified Eagle's medium (DMEM) and Hanks' balanced salt solution (HBSS) were purchased from Invitrogen Life Technologies; Fetal bovine serum (FBS), TNF-α and IL-1β were purchased from BioAbChem Inc. (Ladson, SC). ALDP antibody was from Chemicon International Inc. (Temecula, CA). ALDRP antibody was custom-made from ANASPEC against the mouse 20-residue c-terminal sequence: 722 CKILGEDSVLKTIQTPEKTS 741. 5-LOX antibody was purchased from Cayman Chemical (Ann Arbor, Michigan). Na^+^K^+^ATPase antibody was purchased from Santa Cruz Biotechnology (Santa Cruz, CA). All other antibodies were from Cell Signalling Technology Inc. (MA, USA) unless otherwise mentioned. ECL and nitrocellulose membranes were purchased from Amersham Biosciences. Fatty acid methyl ester (FAME) standards were obtained from Supelco (Bellefonte, Pennsylvania). [l-^14^C] Lignoceric acid was prepared as described earlier [28]. [1-^14^C] Palmitic acid and ^125^I-labeled protein A were obtained from ICN (Cleveland, Ohio).

### Stable Lentiviral vector-mediated gene silencing of Abcd1

A set of 3 human (SK-009605-00-10) and rat (SK-098142-00-10) specific SMART vector 2.0 lentiviral shRNA particles (10^8^ TU/ml) for Abcd1 were purchased from Thermo Fisher Scientific Dharmacon (CO, USA). The vector had an hCMV promoter, a TurboGFP reporter gene and a puromycin selection gene. SMART vector 2.0 non-targeting shRNA control particles (NT) (10^8^ TU/ml, Thermo Fisher Scientific Dharmacon, CO, USA) designed so that no known gene in human, mouse or rat will be targeted were used as negative controls.

Human U87 astrocytes and rat B12 oligodendrocytes were cultured in DMEM with 10% FBS in the presence of antibiotic, and viral particles (Abcd1 and non-targeting control) were added with a multiplicity of infection (MOI) of 2.5 and 3.0 respectively for U87 astrocytes and B12 oligodendrocytes. TurboGFP-expression was analysed using microscopy and GFP-positive cells were selected using puromycin (3.0 µg/ml for U87 astrocytes and 2.0 µg/ml for B12 oligodendrocytes). The selected cells were maintained in culture media containing puromycin at 0.5 µg/ml until further use. Abcd1 silencing was observed by Western blot and mRNA quatification.

### *In vivo* SAHA treatment in Abcd1-knockout (Abcd1-KO) mice

Animal procedure was approved by the MUSC Animal Review Committee, and all animals received humane care in compliance with MUSC's experimental guidelines and the National Research Council's criteria (Guide for the Care and Use of Laboratory Animals).

Abcd1-KO (C57BL6) and control (normal) (C57BL6) breeding pairs were purchased from Jackson Laboratory (Ban Habor, ME, USA) and maintained at the institution's animal facility. The genotypes of all newborns from Abcd1-KO were determined by a PCR-based method as described (Forss-Petter et al. 1997) using the primers suggested in data sheets from Jackson Laboratory (http://jaxmice.jax.org). 5-CACAGCCTCTCTCCTTAAGACC-3 (oIMR1120), 5-CTCGTTGTCTAGGCAACTGG-3 (oIMR1121) and 5-CTTCTATCGCCTTCTTGACG-3 (oIMR1122). Two primers detect disrupted allele Abcd1-KO. Presence of the wild-type allele was revealed in the same reaction by using a third primer (oIMR1121). PCR consisted of a 10 min heating step at 95°C, followed by 35 cycles of denaturation at 95°C for 1 min, annealing at 54°C for 1 min, and extension at 72°C for 1 min. The PCR products were then subjected to agarose gel electrophoresis. Vorinostat or suberoylanilide hydroxamic acid (SAHA) (Cayman chemical, Michigan, USA) therapy was performed on 60–65 day-old Abcd1-KO male mice. Animals were daily treated with SAHA (50 mg/kg body weight) in diet (prepared by Research Diet Inc., New Brunswick, NJ) for 62 days. Brain tissue, (harvested from cortex area) from control (normal), Abcd1-KO and SAHA-treated Abcd1-KO mice was homegenized in RIPA buffer in a ratio of 1∶10 (weight/volume). After centrifugation of samples at 1000 rpm for 2 min to remove all debris, supernatant was collected and protein amount was estimated. 200 µL supernatant (∼1 mg of protein) was used for quantification of VLCFA by GC-MS.

### Fatty Acid β-Oxidation

The peroxisomal oxidation of fatty acids in control, Abcd1-deficient and SAHA-treated Abcd1-deficient astrocytes and oligodendrocytes was determined in 6-well plates as described previously [7]. β-oxidation of fatty acids to acetate (water soluble product) was determined using [1-^14^C]-labeled fatty acids as substrate (C_24∶0_, lignoceric acid or C_16∶0_, palmitic acid (ARC, St. Louis, MO)) as described previously [29]. Cells grown in parallel in same plate were used to determine the protein present in the assays. Experiments were performed in triplicate.

### Lipid Extraction and Fatty Acid Analysis

Total lipids were extracted from control, Abcd1-deficient and SAHA-treated cells as described previously [30]. FAME were analyzed by GC (Shimadzu chromatograph GC-15A attached to a Shimadzu chromatopac C-R3A integrator) using a fused silica capillary column (25 M 007 series methyl silicone, 0.25-mm internal diameter, 0.25-µm film thickness) from Quadrex Corporation (Woodbridge, CT) in a gas chromatograph GC-17A connected with a flame ionization detector from Shimadzu Corporation.

### Preparation of carbonate membranes

Cells were harvested with sucrose buffer (0.25 M sucrose, 1 mM EDTA, 3 mM imidazole buffer, pH 7.4), and subjected to sonication (10 sec at 8–9 watts output power). The homogenate was centrifuged at 2,500 rpm for 5 min to remove unbroken cells. To isolate carbonate membranes (membrane sheets containing integral membrane proteins), equal protein amount from control and X-ALD cell homogenates were diluted with an ice-cold solution of 0.1 M sodium carbonate (pH 11.5) containing 30 mM iodoacetamide. After 30 minutes of incubation at 4°C, carbonate membranes were sedimented by centrifugation at 35,000 rpm for 1 hour in a 70Ti rotor (Beckman-Coulter, Fullerton, CA). The sedimented membranes were washed twice with cold water, lyophilized and stored at −70°C until use.

### Western Blot Analysis

40 µg of total cellular protein was resolved by electrophoresis on 4–20% polyacrylamide gels. After incubation with antiserum raised against ALDP, ALDRP, peroxisomal membrane protein 70 (PMP70), 5-lipoxygenase (5-LOX), B-cell lymphoma 2 (Bcl-2), B cell lymphoma-X long (Bcl-xL), Bcl-2-associated X (Bax), and Bcl-2-antagonist/killer (Bak), Na^+^K^+^ATPase and cleaved caspase-9 and 3, the membranes were then incubated with horseradish peroxidase-conjugated anti-rabbit or mouse IgG for 1 h. The membranes were detected by autoradiography using ECL-plus (Amersham Biosciences) after washing with TBST buffer.

### cDNA Synthesis and Real-time PCR

Following total RNA extraction using TRIzol (Invitrogen) per the manufacturer's protocol, single-stranded cDNA was synthesized from total RNA as described previously [7]. Real time PCR was conducted using Bio-Rad iCycler (iCycler iQ Multi-Color Real Time PCR Detection System; Bio-Rad). Primers for human and rat Abcd1, Abcd2, Abcd3, ELOVL1, and ELOVL3 were purchased from Qiagen. Thermal cycling conditions were as follows: activation of DNA polymerase at 95°C for 10 min, followed by 40 cycles of amplification at 95°C for 30 s and 60°C for 30 s. The normalized expression of target gene with respect to glyceraldehyde-3-phosphate dehydrogenase or 18S RNA was computed for all samples using Microsoft Excel data spreadsheet.

### Organotypic hippocampal slice culture (OHSC)

Hippocampal specimens were derived from three patients submitted to epilepsy surgery at the Erlangen Epilepsy Center. The patients suffered from drug-resistant epilepsy and were scheduled for hippocampal resection. VPA treatment was stopped several weeks prior to surgery. For scientific use of tissue specimens, written informed consent was obtained from each patient with the approval of the local ethics committee of the University Hospital of Erlangen. Surgical specimens were prepared and processed as described for rat OHSCs [31]. After dissection of the frontal pole of the hemispheres and the cerebellum, the brains were cut in 350-µm thick horizontal slices on a vibratome (Leica Microsystems, Wetzlar, Germany). For each experiment, three slices were transferred into culture plate insert membranes (BD Biosciences, San Jose, CA, USA) and thereafter into six-well culture dishes (BD Biosciences) containing 1.2 mL culture medium as described in detail by Eyupoglu et al. [32]. One day after preparation, the culture medium was changed, and OHSCs were exposed to the test compound for 48 h and snap-frozen in liquid nitrogen, as described previously [32].

### Statistical analysis

Using the Student Newman-Keuls test and ANOVA, p values were determined for the respective experiments from three identical experiments using GraphPad software (GraphPad Software Inc. San Diego, CA). The criterion for statistical significance was p<0.05.

## Results

### Stable silencing of Abcd1 in U87 astrocytes and B12 oligodendrocytes using lentiviral-shRNA vector

**Figure 1:**
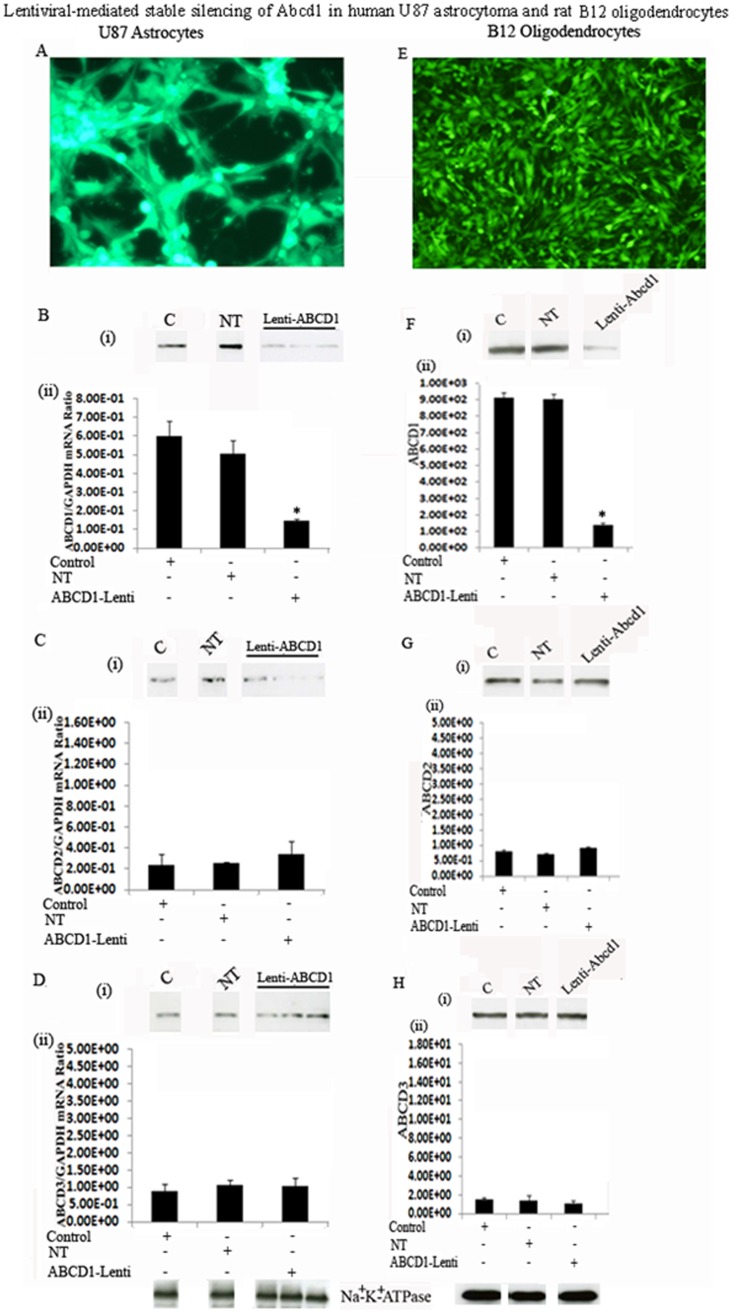
Lentiviral-mediated stable silencing of Abcd1 in human U87 astrocytoma and rat B12 oligodendrocytes. Pool of three GFP-tagged lentiviral-shRNAs for Abcd1 was used for transduction of human U87 astrocytes and rat B12 oligodendrocytes. Human U87 astrocytoma cells (U87-astrocytes) and rat B12 oligodendrocytes (B12 oligodendrocytes) were tranduced with either non-targeting (NT) or Abcd1-Lentiviral (Abcd1-Lenti) particles. Following puromycin selection there was a near-complete selection of transduced cells (**A and E**). Lentiviral-shRNAs induced gene silencing, indicated by a significant decrease in Abcd1 expression, analyzed by Western blot immunoassay detected with monoclonal antibody against Abcd1 protein levels (**B-i and F-i**) and qRT-PCR (**B-ii and F-ii**). Abcd2 (**C and G**) and Abcd3 (**D and H**) protein levels (**i**) and mRNA expression (**ii**) in control, NT and Abcd1-deficient U87 astrocytes (n = 3) and B12 oligodendrocytes. Abcd2 and Abcd3 mRNA levels were determined by quantitative realtime qRT–PCR and normalized to GAPDH. Data are represented as mean±SD. Protein levels of the peroxisomal transporters Abcd1 (**B-i and F-i**), Abcd2 (**C-i and G-i**) and Abcd3 (**D-i and H-i**) were analyzed by Western blot in membranes fractions obtained by carbonate treatment (membrane preparation containing integral membrane proteins), as indicated in Methods section. Na^+^/K^+^-ATPase (plasma membrane protein) was used as indicator of protein loading for plasma membrane fractions. *P<0.001 Abcd1-Lenti compared to control or NT cells.

The observed astrocytic inflammatory activity and demyelination in X-ALD brain [10], [15], [33] suggests that VLCFA-induced inflammatory activity may participate in X-ALD pathology. Therefore, to understand the role of Abcd1 loss in astrocyte and oligodendrocyte functions, we stably silenced the U87 astrocytes and B12 oligodendrocytes for Abcd1 using lentiviral-shRNA. Following transduction, successfully transduced cells were selected with puromycin (3.0 µg/ml for U87 astrocytoma (**Fig. 1A**) and 1.5 µg/ml for B12 oligodendroglia (**Fig. 1E**)). Lentiviral-mediated silencing of Abcd1 in U87 astrocytes (**Fig. 1A**) and B12-oligodendrocytes (**Fig. 1E**) was highly successful (as seen with GFP fluorescence). Subsequently the cultures were maintained in 0.5 µg/ml puromycin. Western analysis using antibody against ALDP on carbonate membranes (membrane preparation containing integral membrane proteins) from control, non-targeting (NT) and Abcd1-deficient U87 astrocytes (**Fig. 1B-i**) and B12 oligodendrocytes (**Fig. 1F-i**) shows almost complete loss of Abcd1 protein in Abcd1-lentiviral silenced cells (**Fig. 1B-i** and **1F-i**). Real time PCR with primers against human and rat ALDP also showed a stable significant (P<0.001) downregulation of Abcd1 expression in U87 astrocytes (**Fig. 1B-ii**) and B12 oligodendrocytes (**Fig. 1F-ii**) respectively. Since overexpression of Abcd2 and Abcd3 can compensate for loss of Abcd1 [34], [35], we investigated the effect of Abcd1 silencing on the expression of Abcd2 and Abcd3 in U87 astrocytes and B12 oligodendrocytes. Abcd1 silencing had no effect on Abcd2 (Fig. **1C** and **1G**) and Abcd3 (**1D** and **1H**) either at protein (**i**) or mRNA (**ii**) levels in U87 astrocytes and B12 oligodendrocytes. This, along with the observation that NT sequence had no effect on levels of Abcd1 as well as Abcd2 and Abcd3 documents that the silencing was specific for Abcd1.

### SAHA induces the Abcd2 and Abcd3 levels in U87 astrocytes and B12 oligodendrocytes stably silenced for Abcd1

**Figure 2:**
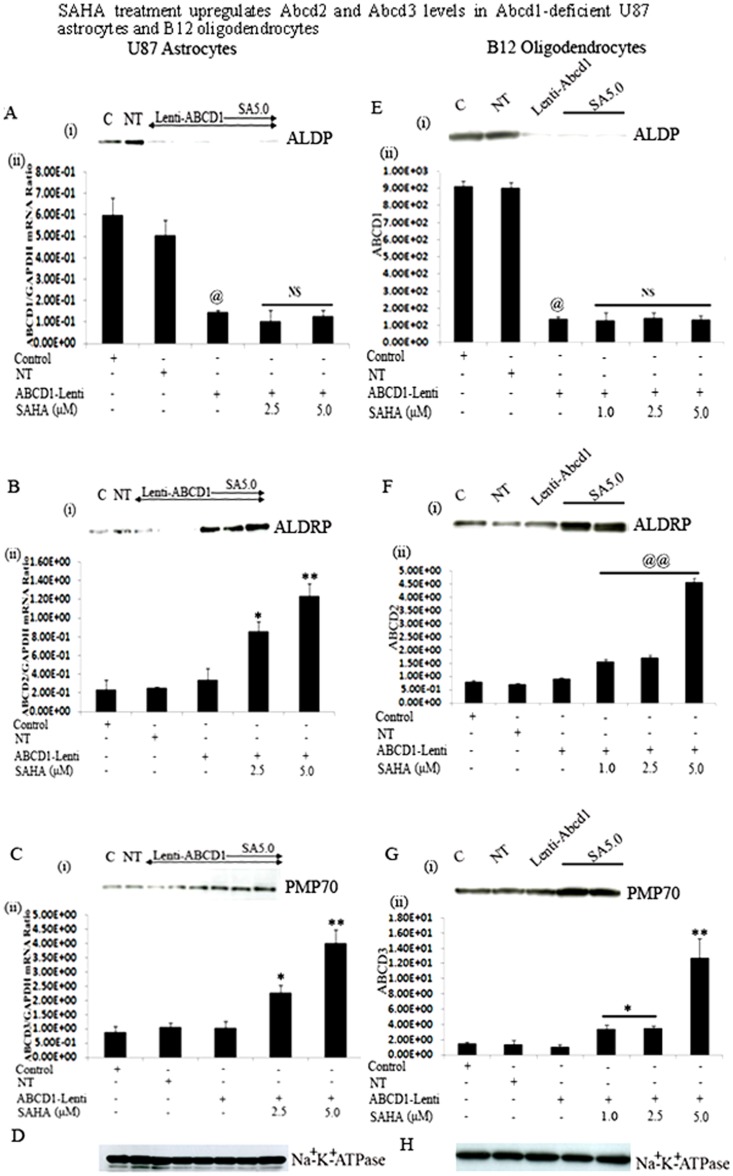
SAHA treatment upregulates Abcd2 and Abcd3 levels in Abcd1-deficient U87 astrocytes and B12 oligodendrocytes. There was no change in Abcd1 protein levels (**A-i and E-i**) and mRNA expression (**A-ii and E-ii**) in Abcd1-deficient U87 astrocytes and B12 oligodendrocytes treated with SAHA. Abcd2 (**B and F**) and Abcd3 (**C and G**) protein levels (**i**) and mRNA expression (**ii**) in control, NT and Abcd1-deficient U87 astrocytes and B12 oligodendrocytes respectively (n = 3) treated with SAHA dose-dependently. Protein levels of the peroxisomal transporters Abcd1 (**A-i and E-i**), Abcd2 (**B-i and F-i**) and Abcd3 (**C-i and G-i**) were analyzed by Western analysis of peroxisomal membrane fractions obtained by carbonate treatment (membrane preparation containing integral membrane proteins), as indicated in Methods section. Na^+^/K^+^-ATPase (plasma membrane protein) was used as indicator of protein loading for plasma membrane fractions. Abcd1 (**A-ii and E-ii**), Abcd2 (**B-ii and F-ii**) and Abcd3 (**C-ii and G-ii**) mRNA levels were determined by qRT–PCR and normalized to GAPDH. Data are represented as mean±SD. ^@^P<0.001 Abcd1-Lenti compared to control or NT cells; **P<0.001 SAHA (5.0 µM) treatment compared to Abcd1-Lenti; *P<0.005 SAHA treatment compared to Abcd1-Lenti; ^@@^P<0.001 SAHA treatment compared to Abcd1-Lenti; NS, non-significant; SA, SAHA.

Abcd2/Aldrp [36] or Abcd3/PMP70 [37], two close Abcd1/Aldp homologues, can compensate for the activity of Abcd1/Aldp. Since the overexpression of Abcd2/Aldrp resulted in complete correction of VLCFA β-oxidation in X-ALD fibroblasts [38] and the prevention of the VLCFA accumulation, axonal degeneration and clinical symptoms in Abcd1 knockout mice [34], Abcd2/Aldrp is an attractive candidate gene for pharmacological gene therapy. Moreover, Abcd2/Aldrp shares a high degree of overlapping biochemical functions regarding the catabolism of VLCFA *in vivo* [39], [40]. We recently demonstrated that SAHA upregulates Abcd2 and Abcd3 expression in human control and X-ALD fibroblasts [35]. Since CNS is the target organ for X-ALD therapy, we therefore further evaluated the therapeutic potential of SAHA in Abcd1-silenced U87 astrocytes and B12 oligodendrocytes. So far, no evidence exists that Abcd1 expression is inducible. Accordingly, SAHA treatment did not induce the protein (**i**) or mRNA (**ii**) levels of residual Abcd1 (if any) in Abcd1-silenced U87 astrocytes (**Fig. 2A**) and B12 oligodendrocytes (**Fig. 2E**). However, treatment with SAHA for 72 h significantly (P<0.001) upregulated the Abcd2 protein (**i**) and mRNA (**ii**) levels in U87 astrocytes (3.6-fold) as well as in B12 oligodendrocytes (0.909±0.047 to 4.57±0.15) ((5.0-fold) in a dose-dependent manner (**Fig. 2B and 2F**). Similarly, protein (**Fig. 2C-i and 2G-i**) and mRNA levels of Abcd3 (**Fig. 2C-ii and 2G-ii**) were significantly increased (P<0.001) in SAHA-treated Abcd1-deficeint U87 astrocytes and B12 oligodendrocytes. Analysis of Na^+^/K^+^-ATPase (plasma membrane protein) levels as indicator of protein loading for membrane fractions indicate no appreciable difference in protein loading between the same cell samples for Western blot studies.

### Induction of Abcd2 and Abcd3 gene expression by SAHA correlates with normalization of fatty acid β-oxidation in Abcd1-silenced U87 astrocytes and B12 oligodendrocytes

**Figure 3:**
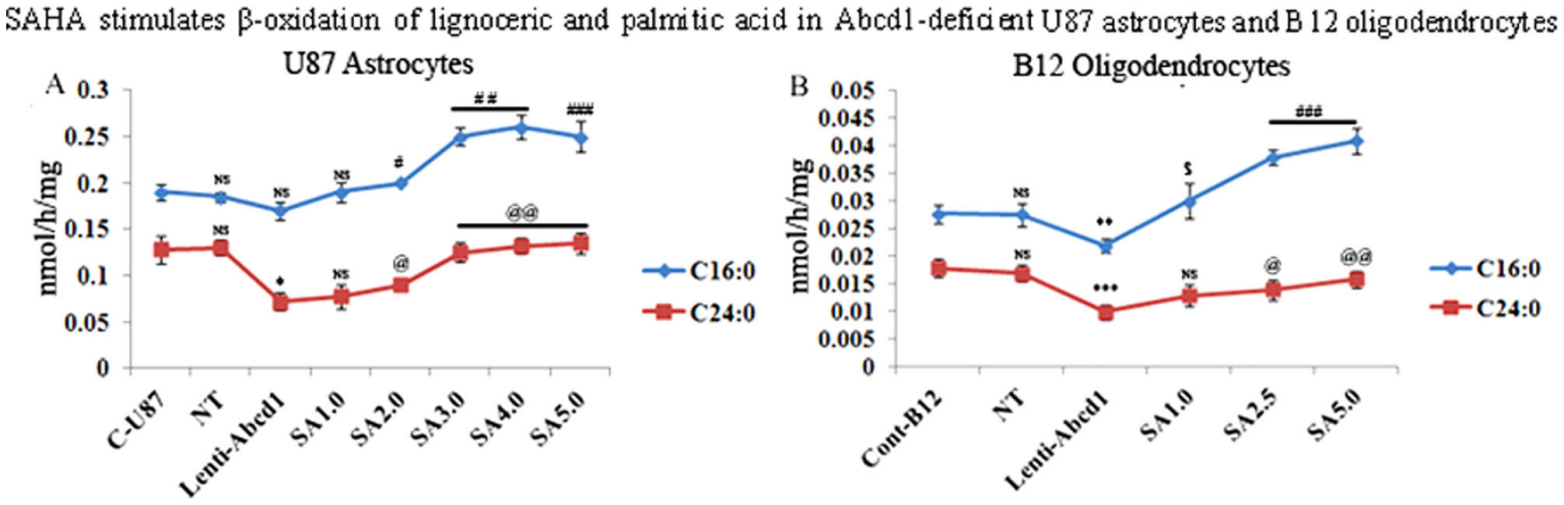
SAHA stimulates the β-oxidation activities of lignoceric and palmitic acid in Abcd1-deficient U87 astrocytes and B12 oligodendrocytes. Palmitic and lignoceric β-oxidation activities were measured in Abcd1-deficient U87 astrocytes (**A**) and B12 oligodendrocytes (**B**) incubated in serum-containing DMEM with different concentrations (µM) of SAHA for 3 days as described in Materials and Methods. After every 24 h, medium was replaced with the addition of fresh reagents. Data are represented as mean±SD. *P<0.002, **P<0.02, ***P<0.005 compared to NT; ^#^P<0.01, ^##^P<0.005, ^###^P<0.001, ^@^P<0.05, ^@@^P<0.005, ^$^P<0.02 SAHA-treated compared to Abcd1-deficient cells; NS, non-significant.

Since mutation/deletion of Abcd1 results in deficient VLCFA β-oxidation [4], we examined these activities in Abcd1-silenced astrocytes and oligodendrocytes with or without SAHA treatment (**Fig. 3A and B**). Using radiolabeled FAs, we observed that the rate of peroxisomal β-oxidation (lignoceric acid) activity was reduced in Abcd1-deficient U87 astrocytes (46%) and Abcd1-deficient B12 oligodendrocytes (41%) as compared to respective control and NT-treated cells (**Fig. 3A–B**). While there was a relatively small decrease (statistically non-significant) in palmitic acid β-oxidation activity in Abcd1-silenced U87 astrocytes (**Fig. 3A**), it was reduced by approximately 20% in B12 oligodendrocytes deficient for Abcd1 (**Fig. 3B**). These observations document that deletion of Abcd1 results in decreased peroxisomal β-oxidation in astrocytes and oligodendrocytes and, interestingly, a partial inhibition of mitochondrial (palmitic acid) oxidation activity in oligodendrocytes.

Overexpression of Abcd2 in whole nervous system or in fibroblasts from Abcd1-deficient mice or from X-ALD patients is shown to restore peroxisomal β-oxidation and to reduce excessive accumulation of VLCFA [34], [38], [40], [41], [42], [43]. Recently we also reported that SAHA treatment increased the lignoceric acid β-oxidation activity in X-ALD fibroblasts [35]. This effect was mediated through induction of Abcd2 while the increase in palmitic acid oxidation by SAHA was independent of Abcd2 expression [7], [35]. Since SAHA upregulated the mRNA expression of Abcd2 and Abcd3 in U87 astrocytes and B12 oligodendrocytes, we next examined the effect of this induction on β-oxidation of lignoceric acid (C24∶0) as well as palmitic acid (C16∶0). SAHA treatment increased the lignoceric acid oxidation in a dose-dependent manner in Abcd1-silenced astrocytes and oligodendrocytes (**Fig. 3A–B**). The highest dose of SAHA (5 µM) at 72 h was found to stimulate lignoceric acid β-oxidation by approximately 45% in U87 astrocytes and by 38% in B12 oligodendrocytes deficient in Abcd1 (**Fig. 3A–B**). SAHA treatment also significantly increased the palmitic acid β-oxidation activities (**Fig. 3A–B**), though maximum activity was reached at day 3 with no further significant increase in oxidation. Increase of both lignoceric and palmitic acids β-oxidation activity in SAHA treated U87 astrocytes and B12 oligodendrocytes deficient in Abcd1 suggests that SAHA treatment has the potential of enhancing fatty acid oxidation in X-ALD brain.

### SAHA reduces the levels of very long chain fatty acids in Abcd1-deficient U87 astrocytes and B-12 oligodendrocytes

**Figure 4:**
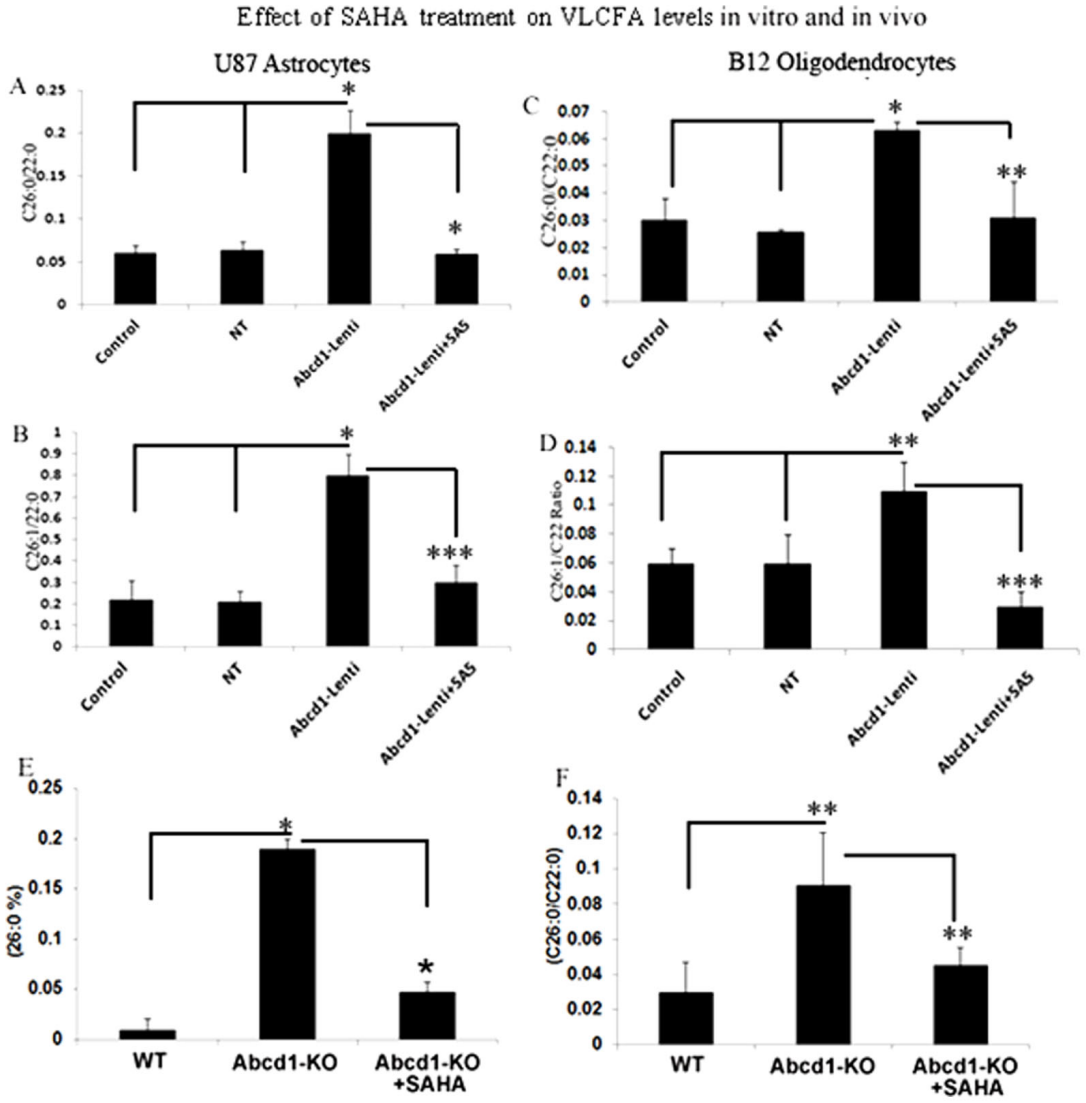
Effect of SAHA treatment on saturated and monounsaturated VLCFA levels in Abcd1-deficient U87 astrocytes and B12 oligodendrocytes. Cells were incubated with serum-containing media with 5 µM SAHA. Fatty acid methyl ester was prepared directly from cells as described in Material and Methods. FAs were analyzed by GC after adding C_27∶0_ as an internal standard. C_26.0_, C_26∶1_, and C_22∶0_ were measured as a percent of total FAs and expressed as ratio of C_26∶0/22∶0_ (**A and C**) and C_26∶1/22∶0_ (**B and D**) in Abcd1-deficient U87 astrocytes (**A and B**) and B12 oligodendrocytes (**C and D**). Results represent the means ±SD of duplicates from three different experiments. C_26∶0_ as percentage of total fatty acids (**E**) and ratio of C_26∶0/22∶0_ (**F**) in wild type (WT), Abcd1-KO and SAHA-treated Abcd1-KO mice. Results represent the means ±SE of duplicates from three different animals. *P<0.001; **P<0.05, ***P<0.005, NT-treated; SA5, SAHA (5 µM).

Dysfunction of ALDP/ABCD1 in X-ALD patients results in decreased VLCFA β-oxidation activity and accumulation of VLCFAs. The C_26∶0_/C_22∶0_ ratio is considered a standard diagnostic tool for the assessment of VLCFA in peroxisomal disorders. Figure 4A shows a 3.1-fold increase in the C_26∶0_/C_22∶0_ ratio in Abcd1-deficient U87 astrocytes and a 3.15 fold increase in Abcd1-deficient B12 oligodendrocytes when compared with their respective control or NT-treated cells. Since SAHA increased the β-oxidation activity of lignoceric acid in Abcd1-deficient U87 astrocytes, we also examined the effect of SAHA on the levels of VLCFA in these cells. As expected a significant (P<0.001) decrease was observed in VLCFA levels in SAHA (5 µM) treated Abcd1-deficient U87 astrocytes as well as B12 oligodendrocytes (**Fig. 4A and C**). These observations document that increased accumulation of VLCFA as a result of Abcd1-deficiency in astrocytes and oligodendrocytes can be attenuated by SAHA treatment.

In addition to saturated VLCFA, there is also accumulation of monounsaturated fatty acids (C26∶1) in plasma, fibroblasts and brain of X-ALD patients as well as in Abcd1-KO mouse indicating that β-oxidation of C26∶1 is also reduced in X-ALD [48], [49]. Accordingly, silencing of Abcd1 also resulted in a significant increase (P<0.001) in levels of C26∶1 in Abcd1-deficient U87 astrocytes and B12 oligodendrocytes (P<0.03) compared to control or NT cells (**Fig. 4B and D**). Higher accumulation of C26∶1 in neural cells likely results in greater oxidative stress in X-ALD brain and Abcd2 was reported to participate in C26∶1 oxidation [20]. In agreement with our previous study using cultured X-ALD skin fibroblasts [35] SAHA treatment (5 µM) also reduced the accumulation (P<0.002) of C26∶1 in Abcd1-deficient U87 astrocytes and B12 oligodendrocytes (**Fig. 4B and D**). These findings document that SAHA treatment corrects the homeostasis of both saturated (C26∶0) and unsaturated (C26∶1) VLCFA in astrocytes.

Next we investigated if SAHA could lower the VLCFA load in CNS of Abcd1-KO mice. VLCFA accumulate differentially between normal looking and diseased areas in CNS of X-ALD patients and levels of VLCFA in CNS correlate positively with disease severity ion human [6], [9], [44]. Abcd1-KO mice were fed SAHA (50 mg/Kg body wt) in diet for 62 days and brains were harvested and VLCFA was quantified in cortex homogenate as described in material and methods. As documented earlier by our laboratory [6], [9] and others [45], [46], [47] VLCFA levels were significantly increased in brains of Abcd1-KO mice. Interestingly, SAHA significantly (P<0.05) reduced the levels of C26∶0 VLCFA in the brain of Abcd1-KO mice (**Fig. 4E and F**). This provides the first preclinical proof-of-concept that SAHA can reduce the VLCFA overload in X-ALD patients.

### SAHA reduces the ELOVL1 and ELOVL3 expression in Abcd1-deficient U87 astrocytes and B12 oligodendrocytes

**Figure 5:**
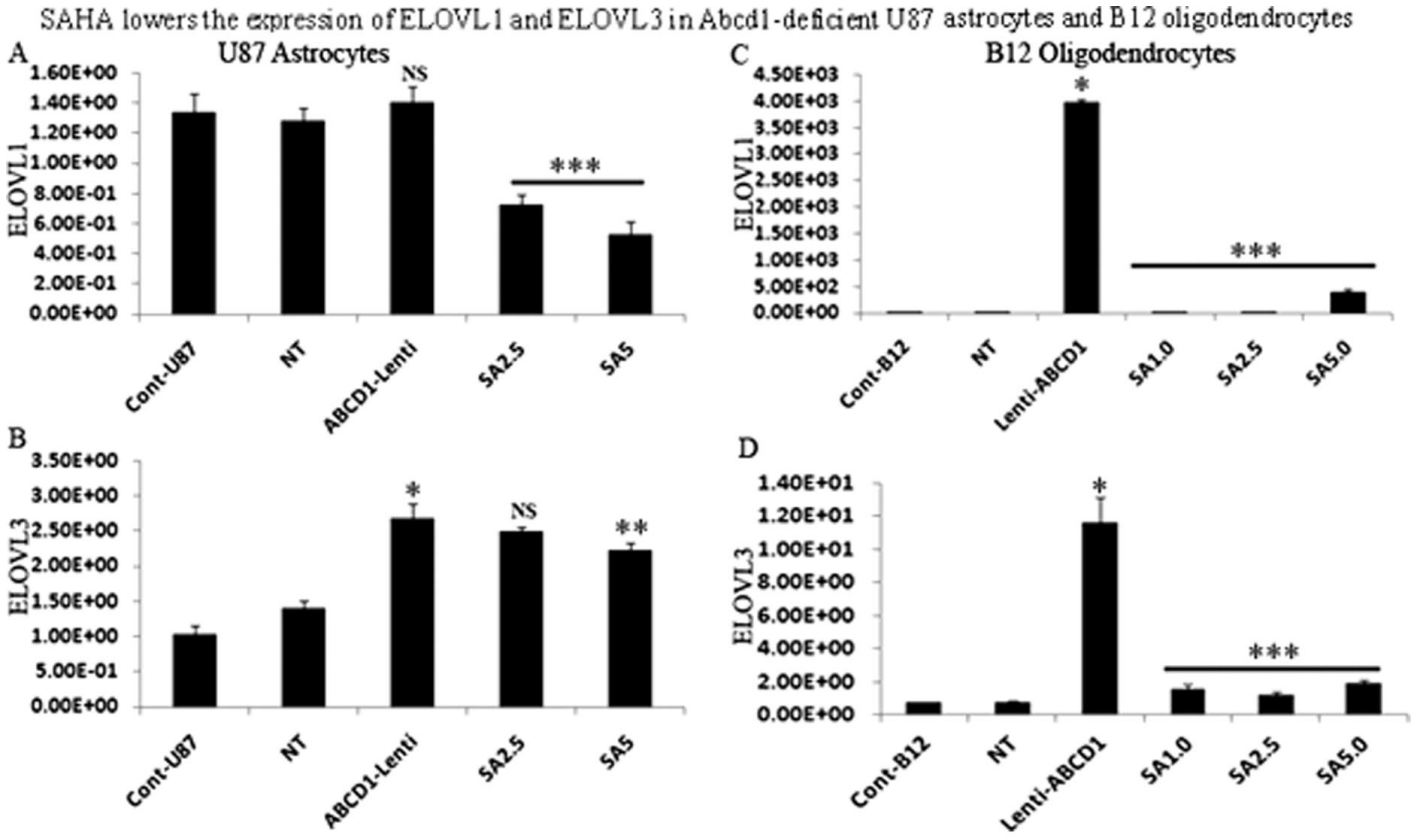
SAHA treatment lowers the mRNA expression of ELOVL1 and ELOVL3 in Abcd1-deficient U87 astrocytes and B12 oligodendrocytes. Abcd1-deficient U87 astrocytes and B12 oligodendrocytes were treated dose-dependently with SAHA for 3 days and mRNA expression of ELOVL1 and ELOVL3 were quantified in control, NT, and untreated/treated Abcd1-deficient U87 astrocytes (**A and B**) and B12 oligodendrocytes (**C and D**) by qRT-PCR normalized to GAPDH. Data are represented as mean±SD. *P<0.001 Abcd1-deficient cells compared with control or NT cells; **P<0.02 SAHA (5.0 µM) treatment compared with untreated Abcd1-deficient U87 astrocytes; ***P<0.001 SAHA treatment compared with untreated Abcd1-deficient cells. NS, non-significant; SA5, SAHA (5 µM).

VLCFAs are produced from LCFAs, provided through diet or generated by fatty acid synthase via fatty acid chain elongation by endoplasmic reticulum membrane-bound enzymes called elongases (ELOVLs), which are believed to perform the first, regulatory, step (condensation) in the elongation cycle. The family of enzymes consists of at least seven members which carry out substrate-specific elongation with fatty acids of different lengths and degrees of unsaturation. ELOVL1 and ELOVL3 have chain length specificity towards VLCFA [50], [51]. Although no significant difference in the levels of ELOVL1 and ELOVL3 in control and X-ALD fibroblasts was observed [18], [35], indicating that availability of substrate is the limiting factor for their elongation, increased expression of ELOVL3 has been reported to be linked to accumulation of VLCFA [20]. Indeed, ELOVL3 expression is elevated in liver of Abcd2 knockout mice, while repressed in liver of Abcd2 overexpressing mice, implying a tight cross-talk between VLCFA synthesis and peroxisomal degradation *in vivo* [52]. Furthermore, knockdown of ELOVL1 in human X-ALD fibroblasts significantly lowers the levels of C26∶0 [18], [20]. Interestingly, ELOVL1 expression was enhanced in oligodendrocytes derived from X-ALD iPSCs [17]. While expression of ELOVL1 remained unaltered, the expression of ELOVL3 was significantly increased (P<0.001) in Abcd1-deficient U87 astrocytes (**Fig. 5A–B**). In rat B12 oligodendroglia deficient for Abcd1 both ELOVL1 and ELOVL3 expression were significantly increased compared to control or NT treated cells (**Fig. 5C–D**). Treatment with SAHA resulted in a dose-dependent decrease in the expression of ELOVL1 and ELOVL3 in Abcd1-deficient U87 astrocytes (**Fig. 5A–B**) while in B12 oligodendroglia maximal inhibition of ELOVL1 and ELOVL3 gene expression was seen at all the doses tested (**Fig. 5C–D**). The results highlight the dual effect of SAHA on induction of β-oxidation activity and inhibition of VLCFA elongation, thus ultimately resulting in a net effect of lowering of the levels of both saturated and unsaturated VLCFAs.

### SAHA inhibits the lipooxidative and the apoptotic response in Abcd1-deficient U87 astrocytes and B12 oligodendrocytes

**Figure 6:**
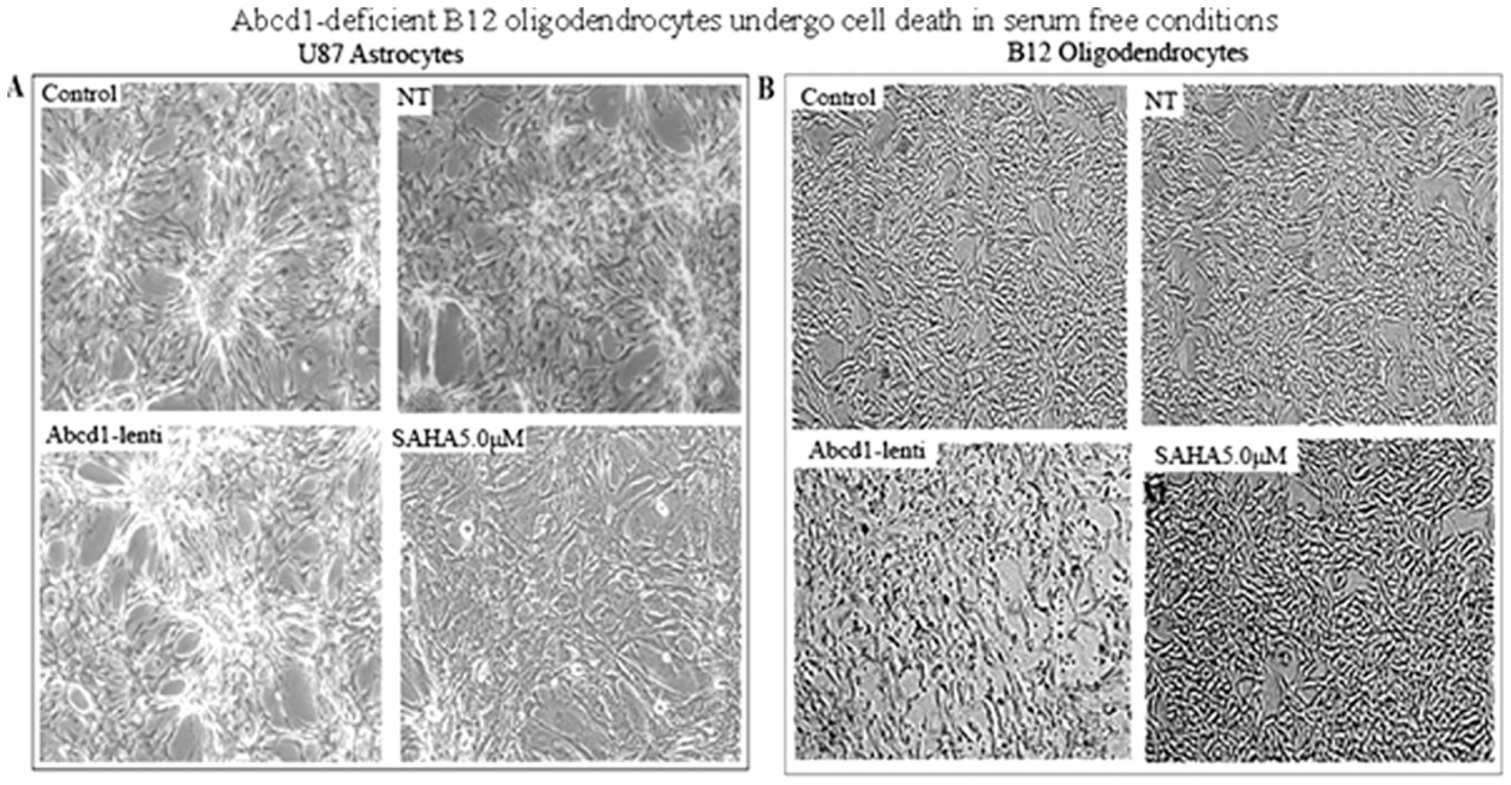
Abcd1-deficient B12 oligodendrocytes undergo cell death in serum free conditions. Phase contrast images of Abcd1-deficient U87 astrocytes (**A**) and B12 oligodendrocytes (**B**) after 24 h in serum-free media with or without SAHA treatment. NT, Non-targeting.

**Figure 7:**
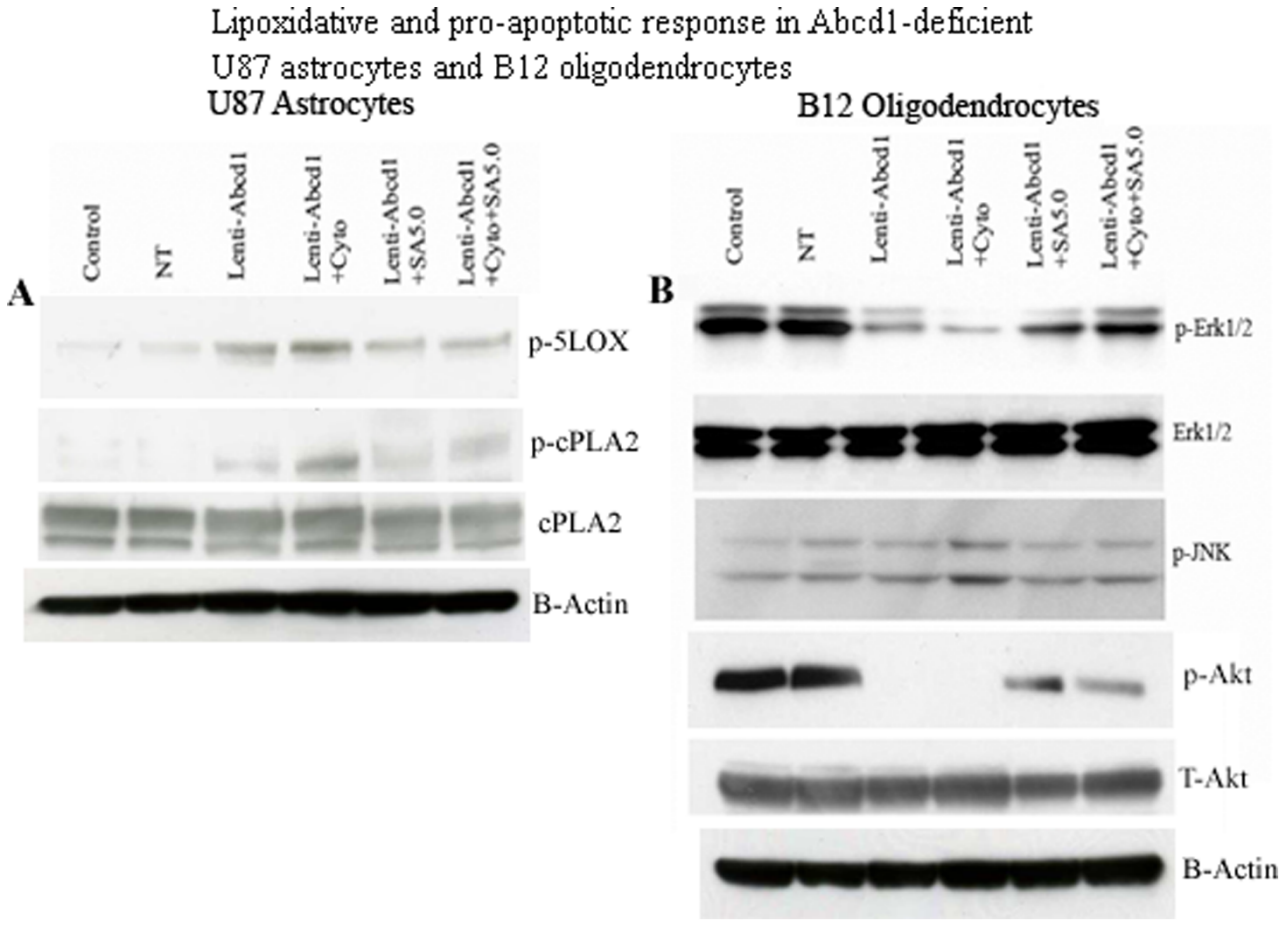
Lipoxidative and pro-apoptotic response in Abcd1-deficient U87 astrocytes and B12 oligodendrocytes is inhibited by SAHA. Cells were treated with cytokines/SAHA in serum-free media and harvested after 24 h. Cell lysate was prepared from control, NT, and untreated/treated Abcd1-deficient U87 astrocytes and B12 oligodendrocytes as described in methods section. (**A**) Representative immunoblots depict the levels of p-cPLA2/cPLA2, p-5LOX and β-actin protein in Abcd1-deficient U87 astrocytes. (**B**) Representative immunoblots depict the levels of p-Erk1/2/Erk1/2, p-JNK, p-Akt/Akt and β-actin protein in Abcd1-deficient B12 oligodendrocytes; SA5, SAHA (5 µM).

Lack of peroxisomes in oligodendrocytes and astrocytes has dramatic consequences on inflammation, demyelination and loss of oligodendrocytes in the CNS [53], [54], [55]. Therefore, it is of interest to examine the effects of Abcd1 deficiency on cell survival/cell death pathways in U87 astrocytes and B12 oligodendrocytes. Phase contrast micrographs of Abcd1-deficient U87 astrocytes maintained in serum free (SF) media shows no effect on cell survival (**Fig. 6A**), while Abcd1-deficient B12 oligodendrocytes when maintained in SF media for 24 h showed enhanced cell death, which was blocked by treatment with SAHA (**Fig. 6B**). Excessive accumulation of VLCFA was reported to cause metabolic alterations leading to membrane perturbation, redox imbalance, and changes in membrane lipid composition [6], [9], [26], as well as the induction of inflammatory mediators in cultured astrocytes [7]. Thus, an appropriate composition of lipids in the cellular membrane is critical for normal function. In astrocytes, altered phospholipid and sphingolipid metabolism in X-ALD [6] paralleled with C26∶0 accumulation and induction of lipooxidative response is mediated by cPLA2/p-cPLA2 and 5-LOX [6], [7]. We therefore examined the expression of lipolytic enzymes cPLA2/p-cPLA2 and 5-LOX in Abcd1-deficient U87 astrocytes. Figure **7A** shows increased immunoreactivity for p-cPLA_2_ and 5-LOX in Abcd1-deficient U87 astrocytes that were inhibited by treatment with SAHA. Western analysis indicated that cell death in Abcd1-deficient B12 oligodendrocytes was mediated by decrease in phosphorylation of Erk1/2 and Akt and the effect was potentiated in Abcd1-deficient B12 oligodendrocytes treated with the cytokine mix that additionally upregulated the JNK pathway (**Fig. 7B**). The dephosphorylation of Erk1/2 and Akt and phosphorylation of JNK was inhibited by SAHA treatment indicating therapeutic potential of SAHA treatment for CNS inflammatory disease of X-ALD.

**Figure 8:**
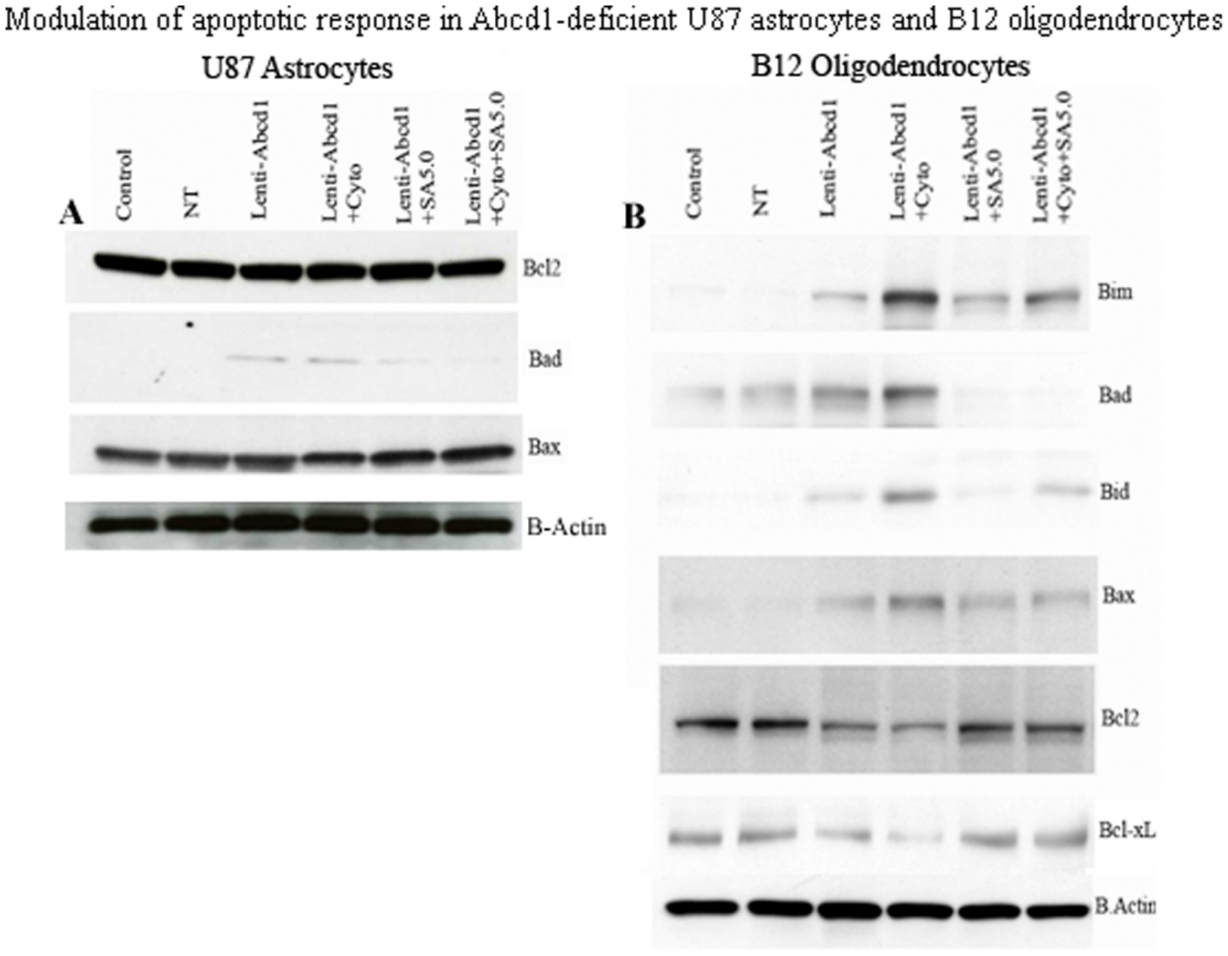
Modulation of apoptotic response in Abcd1-deficient U87 astrocytes and B12 oligodendrocytes. Cells were treated with cytokines/SAHA in serum-free media and harvested after 24 h. Cell lysate was prepared from control, NT, and untreated/treated Abcd1-deficient U87 astrocytes and B12 oligodendrocytes as described in methods section. (**A**) Representative immunoblots depict the levels of Bcl_2_, Bad, Bax and β-actin protein in Abcd1-deficient U87 astrocytes. (**B**) Representative immunoblots depict the levels of Bcl_2_, BcL-xL, Bax, Bad, Bim, Bik and β-actin protein in Abcd1-deficient B12 oligodendrocytes. SA5, SAHA (5 µM).

Mitochondria have key roles in cellular apoptosis, a highly regulated genetic program of cell death. The functional disturbance of mitochondria is critical for cell survival, and exogenous VLCFA treatment has been shown to cause mitochondrial membrane potential changes resulting in cell death [56]. Therefore, we investigated the effect of VLCFA accumulation caused by Abcd1-deficiency on mitochondrial pro- and anti-apoptotic proteins. The ‘commitment’ to the release of proapoptotic factors from the mitochondria depends primarily on the balance between pro- and antiapoptotic members of the Bcl-2 family of proteins; Bcl-2 and Bcl-xL stabilize mitochondrial integrity, while Bax and Bak destabilize this organelle. Binding of Bad to Bcl-xL is thought to cause mitochondrial damage by displacing Bcl-xL and allowing oligomerization of proapoptotic Bax and Bak. There was no change in anti-apoptotic protein (Bcl-2) or proapoptotic protein (Bax) immunoreactivities in Abcd1-deficient human U87 astrocytes (**Fig. 8A**). The only pro-apoptotic protein induced was Bad in Abcd1-deficient astrocytes; no other mitochondrial proapoptotic proteins (Bid, Bim) were induced (data not shown). On the other hand, the observed cell loss in Abcd1-deficient B12 oligodendrocytes prompted the following studies to decipher the molecular mechanism of this cell death. The inhibition of ERK-1/2 activation (**Fig. 7B**) was associated with mitochondrial dysfunction from decreased immunoreactivity for Bcl-xL and Bcl-2 and increased immunoreactivity for Bad, Bid, Bim and pore-forming Bax protein in Abcd1-deficient B12 oligodendrocytes (**Fig. 8B**). Since X-ALD neuropathology is associated with induction of inflammatory mediators [7], [9], [14], cytokines may augment the Abcd1-silencing mediated effects in oligodendrocytes [9]. Accordingly, cytokine treatment (TNF-α (10 ng/ml) + IL-1β (10 ng/ml)) of Abcd1-deficient B12 oligodendrocytes further downregulated cell survival pathways (Erk-1/2) and upregulated proapoptotic proteins (Bax, Bid, Bim and Bad). Treatment of Abcd1-deficient B12 oligodendroglia with SAHA (5 µM) was able to reverse the effects of Abcd1-deficiency by increasing the Erk-1/2 phosphorylation, antiapoptotic proteins (Bcl-2 and Bcl-xL) and decreasing the pro-apoptotic proteins (Bad, Bim, Bax and Bid) levels (**Fig. 8B**). Taken together these observations suggest that peroxisomal dysfunction (loss of Abcd1) in oligodendrocytes predisposes them to an apoptotic response in X-ALD and that SAHA treatment protects against VLCFA-mediated proapoptotic signaling pathways and loss of Abcd1-deficient B12 oligodendrocytes.

### Abcd1-deficiency-initiated apoptosis in U87 astrocytes and B12 oligodendrocytes is associated with proteolytic processing of caspase-9 and caspase-3

**Figure 9:**
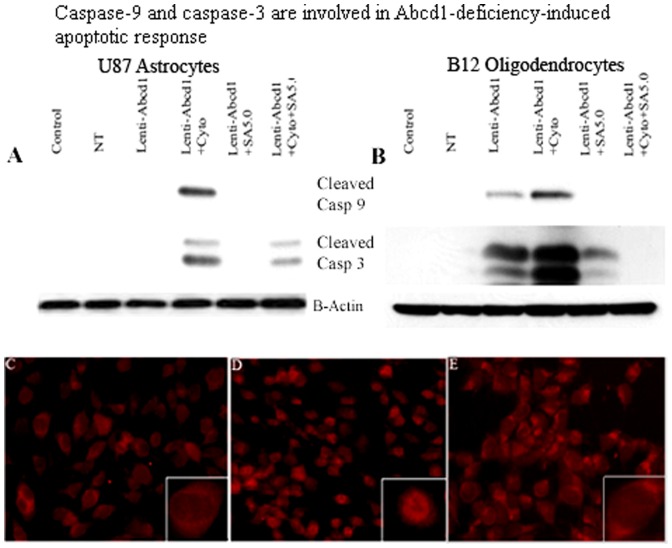
Caspase-9 and caspase-3 are involved in the Abcd1-deficiency-induced apoptotic response. Cells were treated with cytokines/SAHA in serum-free media and harvested after 24 h. Cell lysate was prepared from control, NT, and untreated/treated Abcd1-deficient U87 astrocytes and B12 oligodendrocytes as described in methods section. Representative immunoblots depict the levels of cleaved caspase-9, cleaved caspase-3 and β-actin protein in Abcd1-deficient U87 astrocytes (**A**) and Abcd1-deficient B12 oligodendrocytes (**B**). Propidium iodide staining in Abcd1-deficient B12 oligodendrocytes (**C**), treated with cytokines (**D**), and treated with SAHA (5 µM) + Cytokines (**E**) for 24 h in SF media. SA5, SAHA (5 µM).

Caspases are cysteine proteases that mediate apoptotic cell death. Initiator caspases, such as caspase-9, exist in an inactive monomeric form in the absence of an activation signal. Caspase-9 physically associates with apoptosis activating factor-1 (Apaf-1) to initiate apoptosis. At the same time anti-apoptotic protein BcL-xL has been shown to interact with caspase-9 and Apaf-1, resulting in inhibition of caspase-9 activation [57]. The association of caspase-9 with anti- as well as pro-apoptotic proteins suggests a major role for caspase-9 for control of apoptosis. Caspase-9, when activated through an apoptosome-induced conformational change, further processes the downstream caspases, such as caspase-3, to carry out execution of apoptosis. For this reason, cell lysates from Abcd1-deficient astrocytes and oligodendrocyte cultures and those treated with SAHA were processed for Western analysis using polyclonal antibodies for the cleaved form of caspase-9. In Abcd1-deficient U87 astrocytes cleaved caspase-9 was detected only upon cytokine treatment (**Fig. 9A**). However, cleavage of caspase-9 was significantly increased in Abcd1-deficient oligodendrocytes and was further potentiated by cytokine treatment (**Fig. 9B**). Because caspase-3 is a converging point for different apoptotic pathways [58] and cleaves key proteins involved in the cell structure and integrity upon proteolytic activation [58], [59], [60], [61], we next examined whether this caspase is involved in Abcd1-deficiency-induced apoptosis. Abcd1-deficient cells were analyzed for proteolytic cleavage and activation of caspase-3 by immunoblot analysis with antibody to cleaved caspase-3. In Abcd1-deficient U87 astrocytes no activation of caspase-3 was observed under similar experimental conditions but was induced by cytokine treatment (**Fig. 9A**). However, compared with control and NT cells, Abcd1-deficient B12 oligodendrocytes had higher degree of proteolytic processing of caspase-3 to its active form and the effect was significantly enhanced on treatment with cytokine mix (**Fig. 9B**). SAHA treatment inhibited the proteolytic cleavage of both caspase-9 and caspase-3. Abcd1-deficiency therefore induces caspase-3-mediated apoptosis early in B12 oligodendrocytes while the astrocytes show the apoptotic response only under the inflammatory milieu. Propidium iodide staining for apoptosis in Abcd1-deficient B12 oligodendrocytes showed intercalation in the nuclear material in cytokine treated cells (**Fig. 9D**) but was inhibited in cells pre-treated with SAHA (**Fig. 9E**).

### *Ex vivo* effects of SAHA on ABCD2 gene expression

**Figure 10:**
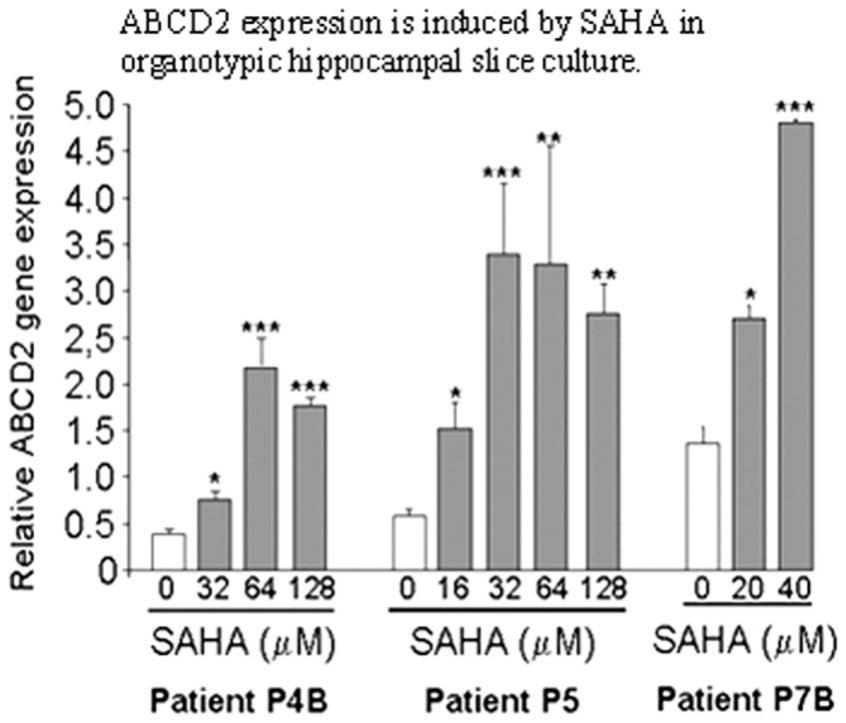
ABCD2 expression is induced by SAHA in organotypic hippocampal slice culture. Human organotypic hippocampal brain slices (n = 3) from patients undergoing surgery for drug-resistant epilepsy were treated with different doses of SAHA for 2 days. ABCD2 expression levels were compared to control tissue by qRT-PCR and normalized to 36B4. Data are represented as mean ±sd. (*P≤0.05, **P≤0.01and ***P≤0.001).

Studies described above document that SAHA treatment attenuates cellular derangements induced by Abcd1 silencing in U87 astrocytes and B12 oligodendrocytes via induction of Abcd2/Abcd3 expression and that SAHA treatment may have therapeutic potential in X-ALD. To further investigate whether SAHA treatment could also induce ABCD2 expression in the adult human hippocampal brain, slices obtained after epilepsy surgery were cultured on the media as described previously [62] and were treated with different doses of SAHA for 48 h. Figure 10 shows that SAHA treatment induced on average a 4-fold increase in ABCD2 gene expression. Cells (**Fig. 1**) versus human tissue (**Fig. 10**) difference may account for the higher doses of SAHA required to achieve the same effect on ABCD2 induction in hippocampal slice cultures.

## Discussion

The exact mechanism that links the Abcd1-loss associated VLCFA excess to axonal degeneration in AMN or to inflammation and demyelination in cALD remains elusive. Cerebral demyelination resulting from activation of astrocytes and death of oligodendrocytes represents the most severe phenotype of X-ALD [5], [55]. We therefore sought to delineate the mechanism of this dramatically opposing response of astrocytes and oligodendrocytes to Abcd1-deficiency-induced VLCFA accumulation in U87 astrocytes and B12 oligodendrocytes stably silenced for Abcd1. *In vitro* studies reported here demonstrate that lentiviral silencing of Abcd1 in human U87 astrocytes and rat B12 oligodendrocytes resulted in reduced β-oxidation and accumulation of VLCFA and, in a novel observation, upregulated ELOVL1 and ELOVL3 to a much greater degree in Abcd1-deficient oligodendrocytes than in Abcd1-deficient U87 astrocytes, suggesting a predominant role for peroxisomal dysfunction in oligodendrocytes contributing to the overall X-ALD neuropathology. Mechanistically, the mitochondrial homeostasis was disturbed in Abcd1-deficient oligodendrocytes and this disturbance was augmented by cytokine treatment. Furthermore, SAHA treatment resulted in the reduction of VLCFA by upregulation of Abcd2/Abcd3 expression and inhibition of ELOVL1 and ELOVL3 in both Abcd1-deficient U87 astrocytes and B12 oligodendrocytes and restored the mitochondrial homeostasis in Abcd1-deficient B12 oligodendrocytes. SAHA also upregulated the ABCD2 expression in human brain hippocampal slice cultures, thus providing a proof-of-principle for use of SAHA for X-ALD neuropathology.

Although the precise function of ALDP is still unknown, the excessive accumulation of VLCFA in X-ALD patients [63] and mice [45], [46], [47], and deficient β-oxidation of these FAs in cells derived from X-ALD patients [4], [64] indicate that it is related to the metabolism of VLCFA in peroxisomes [4], [65]. Accordingly, lentiviral silencing of Abcd1 resulted in up to 99% stable downregulation of mRNA and protein levels for Abcd1 and decreased VLCFA β-oxidation activity in U87 astrocytes and B12 oligodendrocytes and higher accumulation of VLCFA in Abcd1-deficient cells. The association of VLCFA accumulation with neuroinflammatory response [5], [7], [14], and enhanced lipotoxic response mediated by leukotrienes subsequently leading to pathology of inflammatory demyelination characteristic of cALD is documented [6], [7]. Therefore, identification and amelioration of predictive factors for the onset of neuroinflammatory pathology is of critical importance for developing a therapy for X-ALD.

In addition to the abnormality in peroxisomal VLCFA β-oxidation [66], the observed enhanced fatty acid chain elongation activity [67] may also contribute to increased accumulation of VLCFA in X-ALD. ELOVL1 and ELOVL3 are suggested to be involved in elongation of saturated and monounsaturated VLCFA [18], [20], [35] and knockdown of ELOVL1 has been shown to lower the levels of C26∶0 and C26∶1 [18]. ELOVL1 expression and VLCFA accumulation was higher in oligodendrocytes derived from X-ALD iPSCs compared to AMN [17]. Interestingly, ELOVL1 and ELOVL3 expressions were induced many fold more in Abcd1-deficient rat B12 oligodendrocytes than in Abcd1-deficient U87 astrocytes. The results suggest a differential contribution of ELOVL pathway to VLCFA accumulation in astrocytes versus oligodendrocytes and also tend to provide a plausible explanation for the higher levels of VLCFA in white matter of cALD postmortem brain compared to that of control (normal) brain [44].

Exogenous treatment of glial cells with VLCFA results in alterations in mitochondrial function and cell death [56]. However, the mechanism by which Abcd1-deficiency induces oligodendrocyte apoptosis is currently unknown. The Bcl-2 family proteins constitute critical control points in the intrinsic apoptotic pathway. The balance between pro-(Bad, Bid, Bim and Bax) and anti-apoptotic (Bcl-2 and Bcl-xL) members of the Bcl-2 family is critical to control mitochondria-induced apoptosis [68]. The temporal expression profiles of these proteins in response to endogenous accumulation of VLCFA due to Abcd1 loss were investigated individually in astrocytes and oligodendrocytes. While the mitochondrial homeostasis was unaltered in Abcd1-deficient U87 astrocytes (**Fig. 8A**), Abcd1-deficiency alone caused the B12 oligodendrocytes to be more sensitive to mitochondrial dysfunction (**Fig. 8B**) and cell death (**Fig. 7B**). These observations provide the first direct evidence of a possible role of peroxisomal dysfunction in oligodendrocyte loss via VLCFA-mediated mitochondrial perturbations in X-ALD. VLCFA-induced secondary proinflammatory response (TNF-α, IL-1β, and IFN-γ) in X-ALD is believed to result in inflammatory demyelination [10], [12], [69]. Based on the recently proposed three-hit hypothesis [55], inflammation (cytokines) that constitutes the second hit results in mitochondrial dysfunction and a robust pro-apoptotic response (**Fig. 7B**) resulting in oligodendrocyte cell death. DNA damage signaling to the mitochondria results in procaspase-9 activation; caspase-9 then cleaves and activates effector caspase-3, orchestrating downstream apoptotic events culminating in cell death [70]. However, the roles of caspases in Abcd1-deficiency-induced apoptosis of oligodendrocytes and astrocytes have not been studied yet. Here we report that the activation of caspase-9 and caspase-3, and mitochondrial dysfunction in response to Abcd1-deficiency leads to oligodendrocyte loss. The inhibition of apoptosis by treatment with SAHA by maintaining the levels of Bcl-2 and Bcl-xL, establishes the role of mitochondrial apoptotic pathway in oligodendrocyte death and may provide an insight into the mechanisms by which Abcd1-deficiency causes oligodendrocyte loss and demyelination in the CNS of X-ALD patients. The observed activation of caspases in the absence of cell death in Abcd1-deficient U87 astrocytes could be explained by the non-traditional role played by caspases in the cytoskeletal remodelling of activated astrocytes without cell death [71].

Therapeutic options for X-ALD are limited at present. Dietary therapy with Lorenzo's oil is a commonly accepted treatment for asymptomatic X-ALD patients but does not halt disease progression in symptomatic patients [72], [73]. Hematopoitic stem cell transplantation [74] and the recently described lentiviral gene therapy [75] currently apply only to cALD and suffer from the limited time window of opportunity and donor match limitations. Under such circumstances, functional redundancy of Abcd2/Aldrp and Abcd3/PMP70 based on sequence homologies to Abcd1/Aldp has been repeatedly suggested as a therapeutic approach [34]. Indeed, silencing of Abcd2 in addition to Abcd1 causes a greater accumulation of VLCFA that triggered a neuroinflammatory response associated with demyelination in X-ALD [7] and with a more severe axonal degeneration in Abcd1 null mice [34], and overexpression of Abcd2 prevented the accumulation of VLCFA [34], [76] and ameliorated the neurological signs of disease in Abcd1-KO mice [34]. Similarly, overexpression of Abcd3 corrects VLCFA levels *in vitro*, although its inactivation in mouse does not lead to VLCFA accumulation [77]. In agreement with our recent observation in human X-ALD fibroblasts [35], SAHA upregulated Abcd2 gene expression and VLCFA β-oxidation activity in Abcd1-deficient human U87 astrocytes and rat B12 oligodendroglia, and lowered the levels of C26∶0 and C26∶1 in Abcd1-deficient U87 astrocytes in the present study. Treatment with SAHA also reduced both ELOVL1 and ELOVL3 expression in Abcd1-deficient U87 astrocytes and B12 oligodendrocytes and maintained the mitochondrial homeostasis of B12 oligodendrocytes under inflammatory environment. Furthermore, the ability of SAHA to induce ABCD2 gene expression in hippocampal slice cultures from epilepsy patients (Fig. 10) suggests that increased β-oxidation activities and lowering of ELOVLs by SAHA can mediate the overall effect of lowering of VLCFA levels in X-ALD patients, especially in neural cells. Although the levels of VLCFAs in serum or cultured fibroblasts of X-ALD patients appear indistinguishable among patients affected by different disease severity, the *in vivo* amount of VLCFAs in particular cell types or lipid classes, might play a crucial role in the mechanism underlying AMN and cerebral inflammatory ALD [6], [9], [44] In good agreement with this hypothesis, we (and others) [6], [9], [44] reported higher C26∶0 levels in normal appearing white matter of patients with the childhood cerebral phenotype than in those with AMN. Further, we previously described, for the first time, a direct correlation between accumulation of VLCFA and induction of neuroinflammatory response [7]. Also, oxidative stress contributes to the pathogenesis of X-ALD and that excess of VLCFAs plays a role in this process [78]. Thus, there is a good rationale for therapies aiming at lowering VLCFAs in the central nervous system (CNS). VLCFA levels were significantly reduced in the brain of Abcd1-KO mice treated with SAHA (in diet) for 62 days (**Fig. 4E and F**). These data confirm the previous observation that SAHA crosses the blood-brain barrier [79] and provides first preclinical proof-of-concept that SAHA is able to lower the VLCFA levels in CNS of Abcd1-KO mice, the target tissue in human X-ALD pathology.

Although the precise mechanism of CNS degradation in X-ALD patients remains to be elucidated, it seems likely that an attenuation of VLCFA derangements, including those in the CNS, has a potential to help prevent the onset as well as to delay the progression of neuroinflammatory component of the disease. At present, pharmacological induction of Abcd2 expression is the most reasonable and feasible therapeutic approach in X-ALD. HDAC inhibition by SAHA has been demonstrated to be effective in correcting the phenotype of other metabolic diseases including Niemann-Pick type C disease [80] and Gaucher disease [81]. It has also repeatedly been shown to be neuroprotective [82], [83], [84], [85] and anti-inflammatory [86], [87], [88], [89]. Together with these, the capacity of SAHA to cross blood brain barrier [79], induce ABCD2 gene in brain (**Fig. 10**), lower VLCFA levels in Abcd1-KO brain, and good oral bioavailability and safety in long-term treatments in pediatric patients [90], provide a proof-of-principle for use of SAHA in X-ALD therapy.
